# Measurement of laryngeal elevation by automated segmentation using Mask R-CNN

**DOI:** 10.1097/MD.0000000000028112

**Published:** 2021-12-23

**Authors:** Hyun Haeng Lee, Bo Mi Kwon, Cheng-Kun Yang, Chao-Yuan Yeh, Jongmin Lee

**Affiliations:** a Department of Rehabilitation Medicine, Konkuk University School of Medicine and Konkuk University Medical Center, Seoul, Korea; b aetherAI corporation, Taipei, Taiwan; c Center for Neuroscience Research, Institute of Biomedical Science & Technology, Konkuk University, Seoul, Korea.

**Keywords:** laryngeal elevation, Mask region proposal-based convolutional neural network, quantitative measurement, videofluoroscopic swallowing study

## Abstract

The methods of measuring laryngeal elevation during swallowing are time-consuming. We aimed to propose a quick-to-use neural network (NN) model for measuring laryngeal elevation quantitatively using anatomical structures auto-segmented by Mask region-based convolutional NN (R-CNN) in videofluoroscopic swallowing study. Twelve videofluoroscopic swallowing study video clips were collected. One researcher drew the anatomical structure, including the thyroid cartilage and vocal fold complex (TVC) on respective video frames. The dataset was split into 11 videos (4686 frames) for model development and one video (532 frames) for derived model testing. The validity of the trained model was evaluated using the intersection over the union. The mean intersections over union of the C1 spinous process and TVC were 0.73 ± 0.07 [0–0.88] and 0.43 ± 0.19 [0–0.79], respectively. The recall rates for the auto-segmentation of the TVC and C1 spinous process by the Mask R-CNN were 86.8% and 99.8%, respectively. Actual displacement of the larynx was calculated using the midpoint of the auto-segmented TVC and C1 spinous process and diagonal lengths of the C3 and C4 vertebral bodies on magnetic resonance imaging, which measured 35.1 mm. Mask R-CNN segmented the TVC with high accuracy. The proposed method measures laryngeal elevation using the midpoint of the TVC and C1 spinous process, auto-segmented by Mask R-CNN. Mask R-CNN auto-segmented the TVC with considerably high accuracy. Therefore, we can expect that the proposed method will quantitatively and quickly determine laryngeal elevation in clinical settings.

## 1 Introduction

Laryngeal elevation is known to protect the airway through glottic closure as well as provide an approximation of the vestibule and arytenoid cartilage during the swallowing reflex.^[1]^ In addition, bolus transfer from the pharynx to the esophagus is facilitated by the stretched cricopharyngeal muscle, which is provoked by laryngeal elevation.^[2,3]^ With this mechanism, laryngeal elevation is an essential movement for safe and efficient swallowing. Laryngeal elevation has clinical importance because a decrease in laryngeal elevation increases the risk of aspiration. A reduced laryngeal elevation velocity is reportedly an independent indicator of aspiration risk.^[1]^

Because laryngeal elevation is a very rapid reaction that takes approximately 1 s, the degree of laryngeal elevation is difficult to accurately determine during the swallowing process.^[4]^ Physical examination methods, such as visual confirmation or palpation, are commonly used to evaluate laryngeal elevation in clinical settings.^[5]^ However, physical examination is relatively subjective and highly dependent on the experience of the examiners.^[6]^ In addition, although laryngeal elevation is included among the clinical tools that are intended to evaluate swallowing through videofluoroscopic swallowing study (VFSS), no specific method for evaluating laryngeal elevation has been clearly presented.^[7–9]^

By using VFSS, several efforts have been made to quantitatively measure laryngeal elevation more accurately by utilizing the movement of the hyoid bone. Dodds et al reported hyoid displacement by frame-by-frame visual inspection of VFSS recordings in normal population.^[10]^ Ishida et al also measured hyoid movement using coordinates of the hyoid bone by frame-by-frame inspection on VFSS.^[11]^ Nam et al measured laryngeal elevation by tracking the anterior margin of the thyroid cartilage in VFSS images.^[12]^ Lee et al quantitatively measured laryngeal elevation using a hyoid bone semiauto-tracking system in VFSS images.^[13]^ Some studies have measured laryngeal elevation utilizing the thyroid cartilage or larynx-to-hyoid approximation. Kagaya et al measured laryngeal elevation through the movement of the hyoid bone and thyroid cartilage.^[14]^ Leonard et al measured larynx-to-hyoid approximation by measuring the distance between the hyoid bone and thyroid cartilage in each frame of VFSS.^[15]^ However, their methods are time-consuming or difficult to apply in clinical settings since they need frame-by-frame visual inspection on VFSS or equipment operating tracking system. Therefore, among the aforementioned methods for objectively measuring laryngeal elevation, none has achieved widespread usage.

Convolutional neural networks (CNNs) are versatile tools for image classification and segmentation.^[16]^ Mask R-CNN is a variation of region proposal-based CNN (R-CNN), which integrates a framework for region-of-interest localization within the CNN architecture, leading to an end-to-end learning framework with upgraded instance segmentation performance.^[17]^ Mask R-CNN is a fast and accurate tool for object detection and segmentation in natural images.^[17]^ It is also used to localize and track objects in medical images or videos such as in magnetic resonance imaging (MRI) and ultrasonography. Recent studies have used Mask R-CNN for automatic needle localization during ultrasound-guided prostate brachytherapy and automatic needle tracking during MRI-guided intervention.^[18,19]^ A study used Mask R-CNN for automated thyroid nodule detection in ultrasonography.^[20]^

Therefore, in this study, we aimed to propose a potentiality of a neural network model for measuring laryngeal elevation quantitatively using the anatomical structures segmented automatically by Mask R-CNN using VFSS, which is quick to use.

## 2 Materials and methods

We collected 12 video clips using VFSS from 12 patients. The clinical characteristics of the enrolled patients are illustrated in Table 1. VFSS was performed in the following order: (1) dysphagia diet 2 (1/2 pouch of thickener added with 100 ml of water), (2) dysphagia diet 3 (1/3 pouch of thickener added with 100 ml of water), (3) dysphagia diet 4 (1/4 pouch of thickener added with 100 ml of water), and (4) free fluid. Each diet was provided in two quantities (small volume of fluid, 3 ml; large volume of fluid, 7 ml). Only when patients had taken a specific type of fluid at a previous step that they moved to the next step. All fluids were fed using a spoon. Video clips obtained using VFSS have only lateral views. To improve the efficiency of the neural network learning process, we selected the frames that included the swallowing reflex from all frames of the video clips for the respective patients and compiled them. The specifications of the video clips were as follows: 640 × 480 pixels, 30 fps, and MP4 format.

**Table 1:** Clinical characteristics of enrolled patients (n = 12).

Patient no.	Age	Sex	Diagnosis	Chronicity of disease	Underlying diseases
1	84	F	Alzheimer's disease	6 yrs	Hypothyroidism
2	60	M	Traumatic brain injury	5 yrs	Sensorimotor peripheral polyneuropathy
3	45	M	Brain tumor	1 yr	Hypertension and asthma
4	82	F	Lewy body dementia	1 yr	Cerebral infarction, diabetes mellitus, and hypertension
5	71	M	Ischemic stroke	1 mo	Diabetes mellitus
6	76	M	Brain tumor	1 mo	Renal cell carcinoma
7	45	M	Brain tumor	1 yr	Hypertension
8	65	M	Hemorrhagic stroke	14 yrs	–
9	80	M	Ischemic stroke	8 yrs	Vascular dementia
10	55	M	Ischemic stroke	4 mos	–
11	63	M	Hemorrhagic stroke	2 mos	Hypertension, and osteopenia
12	56	F	Neuromyelitis optica	1 mo	–

### 2.1 Data collection

**Figure 1:**
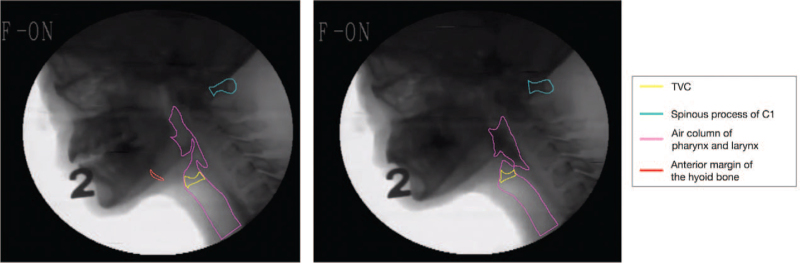
Manually-segmented anatomical structures in two frames of the videofluoroscopic swallowing study videoclips obtained from a patient. TVC = thyroid cartilage and vocal fold complex.

One researcher drew the anatomical structure on the respective video frames using Wacom Cintiq Pro 13 (Wacom Co., Ltd., Otone, Saitama, Japan), which was confirmed by a physician. A closed curve was drawn, which involved the thyroid cartilage and the vocal folds (TV complex, TVC) on one frame in every three frames for efficient learning (Fig. 1). Another closed curve was also drawn, which involved the C1 spinous process, which was used as a reference for measuring laryngeal elevation. In addition, a third closed curve was drawn, which involved the anterior margin of the hyoid bone and airway column of the pharynx and larynx, to improve the efficiency of the Mask R-CNN learning process. Manual segmentation took 6 months (December 3, 2018, to May 31, 2019). We manually segmented 5620 video frames from 12 video clips of the patients.

### 2.2 Data preprocessing and Mask R-CNN architecture

**Figure 2:**
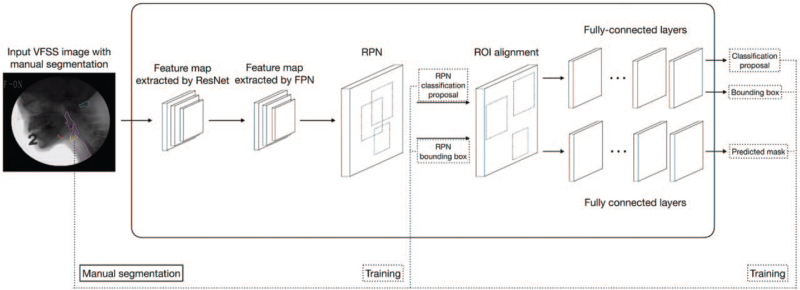
Mask R-CNN architecture. FPN = feature pyramid network, R-CNN = region based convolutional neural network, ROI = region of interest, RPN = region proposal network, VFSS = videofluoroscopic swallowing study.

We collaborated with a Taiwanese AI corporation (aetherAI, Co., Ltd., Taipei, Taiwan) to build a framework of neural networks and training networks. The images of each frame were augmented through random rotation (−30° to 30°), random scale (0.8–1.2), random shift (−10% to 10%), and flip, and we added noise for more efficient learning and avoidance of overfitting. Gaussian blur was used to reduce image noise. The Mask R-CNN architecture includes a deep feature learning network (i.e., ResNet) for feature extraction, a region proposals network for proposal generation, an region of interest (ROI)Align layer for resizing of object proposals, a fully convolutional layer for mask prediction, and a fully connected layer for proposal classification and regression, as illustrated in Figure 2. In this study, ResNet50 was used to extract features for the given image frame. The loss function of Mask R-CNN is defined as the sum of the loss of classification, localization, and segmentation mask as follows: *L* = *L*_*cls*_ + *L*_*box*_ + *L*_*mask*_ where *L*_*cls*_ is the classification loss, which indicates how close the predictions are to the true class, and *L*_*box*_ is the bounding box loss, which illustrates how good the model is at localization. Classification loss *L*_*cls*_ and bounding box loss *L*_*box*_ were defined as in the study conducted by Girshick.^[21]^*L*_*mask*_ is calculated as follows: $L_{mask}=-\frac{1}{m^{2}}\sum_{1\leq i,j\leq m}\left[y_{ij}Log\;{\overset{\circ }{y}}_{ij}^{k}+(1-y_{ij})\;Log(1-{\overset{\circ }{y}}_{ij}^{k})\right]$ which was defined as in the study conducted by He et al.^[17]^ Losses of classification, localization, and segmentation were equally weighted.

### 2.3 Training Mask R-CNN

The dataset was split into 11 videos (4686 frames) for model development and one video (532 frames) for testing the derived model. An 11-fold cross-validation was employed for tuning the hyperparameters of Mask R-CNN. The learning rate was 0.001 and the number of steps per epoch was 800. Parameter regularization was also utilized through L2-regularization of 0.0001. Details of Mask R-CNN hyperparameters are presented in Table 2. Early stopping was used to avoid overfitting of a derived model.

**Table 2:** Hyperparameters of Mask R-CNN.

Hyperparameter	Values
Backbone network architecture	ResNet50
Backbone stride	[4, 8, 16, 32, 64]
Bounding box refinement standard deviation for final detection	[0.1, 0.1, 0.2, 0.2]
Maximum number of final detections	4
Minimum probability value to accept a detected instance	0.6
Non-maximum suppression threshold for detection	0.5
Size of the fully connected layers in the classification graph	1024
Gradient norm clipping	5.0
Learning momentum	0.9
Learning rate	0.001
Mask pool size	14
Mask shape	[28, 28]
Maximum number of ground truth instances	2
Pool size	7
Post number of ROI inference	512
Post number of ROI training	512
ROI positive ratio	0.33
RPN anchor ratio	[0.5, 1, 2]
RPN anchor scale	[8, 16, 32, 64, 128]
RPN anchor stride	1
RPN bounding box refinement standard deviation	[0.1, 0.1, 0.2, 0.2]
RPN NMS threshold	0.7
RPN train anchors per image	16
Steps per epoch	800
Top-down pyramid size	256
Train batch normalization layers	False
Train ROIs per image	20
Use mini mask	True
Use RPN ROIs	True
Validation steps	100
Weight decay	0.0001

NMS = non-maximum suppression, R-CNN = region based convolutional neural network, ROI = region of interest, RPN = region proposal network.

### 2.4 Validation of the model

We checked the validity of the trained model by using the intersection over union (IoU), which indicated an overlapped lesion of the ground truth (manually segmented region) and automatically segmented region over a union of them.

$IoU=\frac{area(S_{m}\cap S_{a})}{area(S_{m}\cup S_{a})}$ (S_m_, manually segmented region; S_a_, automatically segmented region by Mask R-CNN).

The overall segmentation performance was evaluated by recall.


Recall=TPTP+FN=true object detectionall ground truth boxes

There were no absolute criteria for IoU for checking the accuracy of segmentation, but it is generally set to 0.5. However, as the purpose of this study was to measure laryngeal elevation, not segmentation itself, we used 0.3 IoU as the cut-off criterion for IoU. A true positive corresponded to the correct detection of the TVC with IoU ≥ 0.3, whereas a false negative corresponded to the detection of the TVC with IoU < 0.3. We used two GPUs (Nvidia GeForce GTX-1080Ti; Nvidia Corp, Santa Clara, CA) to train the model. TensorFlow 1.14.0 and Keras 2.2.4 packages in Python 3.7.3 were used to implement Mask R-CNN (Fig. 2).

## 3 Results

**Figure 3:**
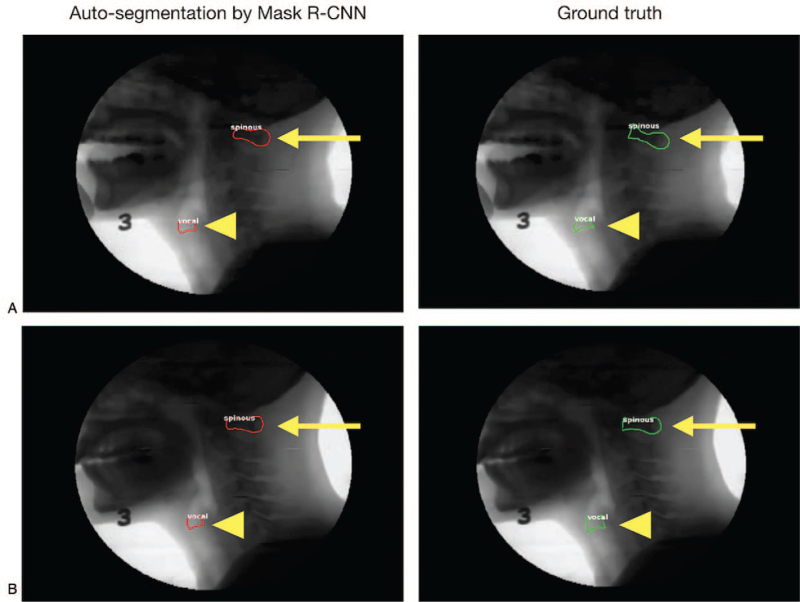
Auto-segmentation of the C1 spinous process and the TVC. (A) An example with a high IoU value. (B) An example with a low IoU value red line, auto-segmentation by Mask R-CNN; green line, ground truth (manually segmented structure); arrowheads, TVC; arrows, C1 spinous process. IoU = intersection over union, R-CNN = region based convolutional neural network, TVC = thyroid cartilage and vocal fold complex.

**Figure 4:**
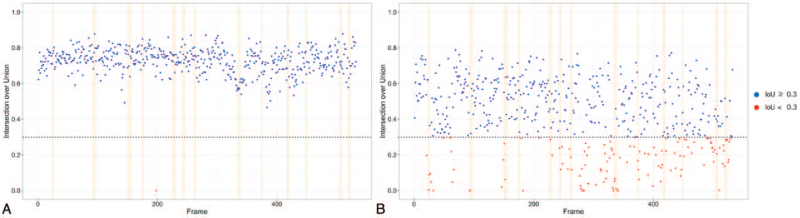
Scatter plot of the IoU by frame. (A) IoU for the auto-segmented C1 spinous process. (B) IoU for the auto-segmented TVC black-dotted line, IoU = 0.3; orange lines, frames when the TVC reaches the peak points. IoU = intersection over union, TVC = thyroid cartilage and vocal fold complex.

The mean IoU values of the C1 spinous process and the TVC were 0.73 ± 0.07 [range, 0–0.88] and 0.43 ± 0.19 [range, 0–0.79], respectively. Examples of the auto-segmentation of the C1 spinous process and TVC are demonstrated in Figure 3. Our results revealed that the IoU of the TVC auto-segmentation decreases when the TVC reaches the highest position, as illustrated in Figure 4B (IoU at the lower point vs. IoU at the peak point, 0.44 ± 0.19 vs. 0.38 ± 0.19; *P* = .010). The IoU of the C1 spinous process and the TVC at each frame are presented in Figure 4A and B, respectively. The recall for the auto-segmentation of the TVC and C1 spinous process by Mask R-CNN were 86.8% and 99.8% (Table 3), respectively. The trained model missed the C1 spinous process only in one frame over 532 frames and the TVC in 70 frames over 532 frames, which means that the IoU was <0.3.

**Table 3:** Test results of automatic segmentation results.

Ground truth	Automatic segmentation of the TVC	Automatic segmentation of the spinous process
Manually segmented TVC	86.8% (462/532)	–
Manually segmented spinous process	–	99.8% (531/532)

TVC = thyroid cartilage and vocal fold complex.

**Figure 5:**
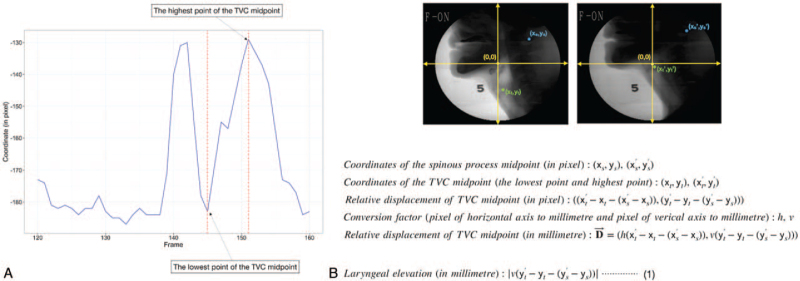
Process of measuring laryngeal elevation. (A) *y*-axis coordinates of the TVC midpoint relative to the C1 spinous process midpoint in pixel. (B) Formula for measuring laryngeal elevation red-dotted line, interval of frames with the longest trajectory of the relative *y*-axis coordinates of the TVC midpoint. TVC = thyroid cartilage and vocal fold complex.

The *y*-axis coordinates of the midpoint of the TVC were plotted relative to the midpoint of the C1 spinous process, and the frames with the longest trajectory were selected (presented as red-dotted line in Fig. 5A). We also confirmed that all the IoU of the TVC occurring in the selected trajectory was >0.3. To calculate the laryngeal elevation, we used the coordinates of the auto-segmented midpoints of the C1 spinous process and TVC in two frames, in which the larynx stayed at the lowest point and reached the highest point. The coordinates in VFSS are in pixels; therefore, we used the conversion factor to convert pixel units to millimeters. The conversion factor for the pixels in the horizontal and vertical axes was obtained using the actual distance of C3 and C4 vertebral body diagonal lengths in the MRI of patients whose VFSS was the test dataset. The formula used to calculate the laryngeal elevation is presented in Figure 5B. Using this formula, the value of laryngeal elevation using the auto-segmented TVC for one swallowing event in the test VFSS dataset was calculated as 35.1 mm, which was similar to that calculated using the manually segmented TVC (37.2 mm).

## 4 Discussion

The TVC in the video clip obtained using VFSS was auto-segmented using Mask R-CNN with a considerable accuracy. However, when the TVC was at the highest point during laryngeal elevation, the IoU value of the TVC fold decreased, while that of the C1 spinous process did not. This might be attributed to the fact that the contour of the TVC becomes indistinguishable from the upper structure of the larynx at the peak point because the laryngeal vestibule is closed, and the margin of the TVC is less demarcated than the C1 spinous process in fluoroscopy images. In addition, the IoU level of auto-segmentation in this study was lower than that of auto-segmentation using MRI or ultrasonography in previous studies.^[19,20]^ This is inevitable because the spatial resolution of fluoroscopy images is lower than that of MR or ultrasound images, which results in the lower contrast of the object in VFSS. In addition, we used the VFSS videoclips with 640 × 480 pixels in this study and, therefore, the boundaries of the structures in the VFSS frame were unclear. To overcome this limitation, we need more training datasets of VFSS with a higher resolution.

Although previous studies have not considered the movement of the vocal folds to assess laryngeal elevation, in this study, we used the movement of the thyroid cartilage and vocal folds as indicators of laryngeal elevation. Terk et al considered the larynx-to-hyoid approximation as an indicator of laryngeal elevation and demonstrated that tracheostomy did not cause significant changes in the dynamics of laryngeal elevation.^[22]^ Kagaya et al measured laryngeal elevation through the movement of the hyoid bone and thyroid cartilage,^[14]^ and Huang et al measured laryngeal elevation through the movement of the hyoid bone and thyroid cartilage and hyoid-larynx approximation on ultrasonography.^[3]^ During laryngeal elevation, the hyoid bone moves upward and anterior, and the larynx approaches the hyoid bone.^[23]^ During the movement of those structures, the vocal folds and arytenoid cartilage attached to the vocal folds are also elevated. Logemann et al stated that the degree of displacement of the arytenoid cartilage was greater than that of the thyroid cartilage during laryngeal elevation.^[24]^ Therefore, the actual laryngeal movement can be accurately evaluated by considering not only the prominence of the thyroid cartilage, which is the anterior margin of the larynx, but also the movement of the vocal fold and arytenoid cartilage, which are the posterior structures. In this study, we used the movement of the thyroid cartilage and vocal folds as indicators of laryngeal elevation since we were not able to draw the contour of the arytenoid as a distinct structure. However, we consider that this method is a good approximation for measuring the degree of laryngeal elevation because the arytenoid cartilage is attached to the vocal folds. This approximation has some weak points. These structures can individually move different distances in diverse trajectories in three dimensions, particularly when pathology is present. Using the mid-point of the TVC is an option, but it would not present alteration in the laryngeal tilt wherein the midpoint of the structures is relatively still, but the thyroid cartilage rotated significantly forward and anteriorly with the arytenoids also elevating while the vocal fold is less elevated overall.

Through the results of this study, we have established the basis for the quantitative assessment of laryngeal elevation by auto-segmentation of the TVC. It has a potentiality as quick-to-use method for quantitative assessment of laryngeal elevation, which is essential for measuring corresponding laryngeal elevation from large-scale data. We expect that the degree of laryngeal elevation can be confirmed rapidly from a large amount of data by using this neural network model. Further, we can set up reference values for laryngeal elevation with better accuracy after training a neural network model using large-scale data. In addition, we can perform motion analysis of laryngeal elevation through our auto-segmentation system, in which we track laryngeal elevation and measure the velocity of laryngeal elevation.

In this study, we obtained the absolute length of laryngeal elevation through the auto-segmentation of the TVC using MRI and mathematical formulas, which may result in the augmentation of measurement error in some cases. In future studies, we need to measure the absolute length using either the distance from the tragus to the angle of the mandible or the size of the coin attached during VFSS. In addition, because the three-dimensional motion is seen in two dimensions in VFSS, we could measure laryngeal elevation only in two dimensions. However, this does not compromise the accuracy of laryngeal elevation measurement because most of the movements due to laryngeal elevation proceed in a two-dimensional plane.

In future studies, we will train the developed model with more labeled data to improve the accuracy of the model and perform correlation study with clinical scales, such as the American Speech-Language-Hearing Association's National Outcomes Measurement System and the penetration-aspiration scale.

In conclusion, we demonstrated that the Mask R-CNN auto-segmented the TVC with considerably high accuracy. We also calculated laryngeal elevation using the midpoint of the TVC and C1 spinous process auto-segmented using Mask R-CNN. Thus, we expect to measure laryngeal elevation quantitatively and quickly in clinical settings.

## Author contributions

Conceptualization, HHL and JML; methodology, BMK, CKY, and CYY; writing – original draft, HHL; supervision, JML. All authors have read and agreed to submit the manuscript.

**Conceptualization:** Hyun Haeng Lee, Jongmin Lee.

**Methodology:** Bo Mi Kwon, Cheng-Kun Yang, Chao-Yuan Yeh.

**Supervision:** Jongmin Lee.

**Writing – original draft:** Hyun Haeng Lee.
